# Long Non-coding RNA GAS5 Regulates T Cell Functions via miR21-Mediated Signaling in People Living With HIV

**DOI:** 10.3389/fimmu.2021.601298

**Published:** 2021-03-12

**Authors:** Lam Ngoc Thao Nguyen, Lam Nhat Nguyen, Juan Zhao, Madison Schank, Xindi Dang, Dechao Cao, Sushant Khanal, Bal Krishna Chand Thakuri, Zeyuan Lu, Jinyu Zhang, Zhengke Li, Zheng D. Morrison, Xiao Y. Wu, Mohamed El Gazzar, Shunbin Ning, Ling Wang, Jonathan P. Moorman, Zhi Q. Yao

**Affiliations:** ^1^Center of Excellence in Inflammation, Infectious Disease and Immunity, James H. Quillen College of Medicine, East Tennessee State University, Johnson City, TN, United States; ^2^Division of Infectious, Inflammatory and Immunologic Diseases, Department of Internal Medicine, Quillen College of Medicine, East Tennessee State University (ETSU), Johnson City, TN, United States; ^3^Hepatitis C Virus/Hepatitis B Virus/Human Immunodeficiency Virus (HCV/HBV/HIV) Program, Department of Veterans Affairs, James H. Quillen VA Medical Center, Johnson City, TN, United States

**Keywords:** Gas5, HIV, lncRNA, miR-21, T cell dysregulation

## Abstract

T cells are critical for the control of viral infections and T cell responses are regulated by a dynamic network of non-coding RNAs, including microRNAs (miR) and long non-coding RNAs (lncRNA). Here we show that an activation-induced decline of lncRNA growth arrest-specific transcript 5 (GAS5) activates DNA damage response (DDR), and regulates cellular functions and apoptosis in CD4 T cells derived from people living with HIV (PLHIV) via upregulation of miR-21. Notably, GAS5-miR21-mediated DDR and T cell dysfunction are observed in PLHIV on antiretroviral therapy (ART), who often exhibit immune activation due to low-grade inflammation despite robust virologic control. We found that GAS5 negatively regulates miR-21 expression, which in turn controls critical signaling pathways involved in DNA damage and cellular response. The sustained stimulation of T cells decreased GAS5, increased miR-21 and, as a result, caused dysfunction and apoptosis in CD4 T cells. Importantly, this inflammation-driven T cell over-activation and aberrant apoptosis in ART-controlled PLHIV and healthy subjects (HS) could be reversed by antagonizing the GAS5-miR-21 axis. Also, mutation of the miR-21 binding site on exon 4 of GAS5 gene to generate a GAS5 mutant abolished its ability to regulate miR-21 expression as well as T cell activation and apoptosis markers compared to the wild-type GAS5 transcript. Our data suggest that GAS5 regulates TCR-mediated activation and apoptosis in CD4 T cells during HIV infection through miR-21-mediated signaling. However, GAS5 effects on T cell exhaustion during HIV infection may be mediated by a mechanism beyond the GAS5-miR-21-mediated signaling. These results indicate that targeting the GAS5-miR-21 axis may improve activity and longevity of CD4 T cells in ART-treated PLHIV. This approach may also be useful for targeting other infectious or inflammatory diseases associated with T cell over-activation, exhaustion, and premature immune aging.

## Introduction

HIV/AIDS pandemic affects 1.1 million individuals in the United States and more than 36 million people worldwide (1, 2). The hallmark of HIV/AIDS is a gradual depletion of CD4 T cells, with progressive decline of host immunity, leading to an increased susceptibility to opportunistic infections, malignancies, and ultimately death (3, 4). While combined antiretroviral therapy (ART) can effectively control viral replication in the majority of people living with HIV (PLHIV), ART does not always result in complete recovery of CD4 T cells (5, 6). Even with satisfactory salvage of CD4 T cell numbers, ART-controlled PLHIV often exhibit immunologic scarring and residual inflammation, inducing an inflammaging phenotype that is characterized by shortened telomeres, poor proliferative capacity, and blunted vaccine responses (7–10). The inflammaging imposed by latent HIV infection exposes the immune system to unique challenges that lead to profound T cell exhaustion and senescence, a major driver of increased infections, cancers, cardiovascular diseases, and neurodegeneration, and similar to what is observed in the elderly (11, 12). Therefore, it is fundamentally important to elucidate the mechanisms underlying T cell aging in PLHIV with ART-controlled latent infection.

In addition to protein-coding genes, the human genome expresses different classes of non-coding RNAs, including microRNAs (miRNA) and long non-coding RNAs (lncRNA) (13–16). Overall, protein-coding genes account for only 2% of the human genome, whereas the vast majority of gene transcripts are non-coding RNAs. MiRNAs (~20 nucleotides) and lncRNAs (>200 nucleotides) are RNA transcripts that do not encode proteins but possess regulatory functions. Using RNA sequencing and annotation of the GENECODE project (17), recent studies revealed over 30,000 lncRNAs (listed at www.noncode.org and www.lncipedia.org), and this number continues to grow, but the functions of these lncRNAs remain largely elusive.

The growth arrest-specific transcript 5 (GAS5) is a lncRNA originally isolated from murine NIH 3T3 cells, and later identified in human cells (NR_002578 and AF141346) (18). GAS5 is induced under stress conditions such as serum starvation and cell-cell contact inhibition and has been reported to possess tumor-suppressive functions in humans (19–24). GAS5 has also been shown to inhibit tumor growth by regulating miR-21 (19, 24), a miRNA that regulates inflammation and immune response (25–28). Of note, the expressions and functions of lncRNAs, as well as miRNAs, are species-, cell-, and disease-specific. In addition to suppressing cancer development and metastasis in humans, GAS5 has been shown to control HIV replication through interaction with miR-873 (29), but its role in regulating T cell functions in ART-controlled HIV infection remains unclear.

We have previously shown that PLHIV exhibit CD4 T cell exhaustion, senescence, apoptosis, and dysfunction, despite ostensibly complete control of viral replication by ART (30–36). How T cell functions are dysregulated in ART-controlled PLHIV is incompletely understood. In this study, we observed differential regulation of GAS5 and miR-21 expressions in CD4 T cells derived from ART-controlled PLHIV and age-matched healthy subjects (HS). Notably, GAS5 controlled miR-21 expression and regulated signaling molecules involved in DNA damage and cellular responses following T cell receptor (TCR) stimulation. Importantly, interrupting this GAS5-miR-21 axis during TCR activation reversed T cell dysfunction and rescued apoptosis in CD4 T cells derived from ART-treated PLHIV. Our data suggest that GAS5 regulates TCR-induced activation and apoptosis of CD4 T cells during HIV infection through a miR-21-mediated signaling. However, T cell exhaustion during HIV infection may be mediated by a mechanism beyond the GAS5-miR-21-mediated signaling. This study provides a novel strategy to improve immunotherapies and vaccine responses in the setting of latent HIV infection.

## Materials and Methods

### Subjects

The study protocol was approved by the institutional review board (IRB) of East Tennessee State University and James H. Quillen VA Medical Center (ETSU/VA IRB; Johnson City, Tennessee). All participants were adults and signed informed consent forms. Participants included 142 people living with HIV (PLHIV) and 58 age-matched HS. All PLHIV participants were on ART, virologically suppressed as evidenced by undetectable (<20 copies/ml) viral RNAs. Blood from HS was supplied by Physicians Plasma Alliance (PPA, Gray, TN) and was negative for HBV, HCV, and HIV infections. The demographic information of all participants is listed in Table 1.

**Table 1:** Demographic information of the study participants.

	**Total**	**Gender male/female**	**Age mean (SD/range)**	**CD4 count mean (SD/range)**
HS	58	46/12	43 (9/34)	N/A
PLHIV	142	122/20	48 (9/47)	987 (389/2,245)

*This table consists of demographic information of the participants in this study, including the total number of subjects, gender (M/F), age (SD/Range), and CD4 number (SD/Range) of each group, either HS or PLHIV*.

### Cell Isolation and Culture

PBMCs were isolated from whole blood of HS and PLHIV using Ficoll-Paque solution (GE Healthcare; Piscataway, NJ) and density gradient centrifugation separation. CD4 T cells were purified from PBMCs using CD4 T cell negative selection kit (Miltenyi Biotec; Auburn, CA). The cells were cultured for 24 h in RPMI-1640 media (Fisher Scientific; Pittsburgh, PA) supplemented with 10% heat-inactivated fetal bovine serum (Atlanta Biologicals; Flowery Branch, GA), 100 IU/ml penicillin, and 2 mM L-glutamine (Thermo Scientific; Logan, Utah) at 37°C and 5% CO_2_ atmosphere. For TCR stimulation, CD4 T cells were cultured in media containing Dynabeads coated with human T-activator CD3/CD28 (Fisher Scientific). For knockdown and overexpression experiments, the cells were stimulated with soluble CD3/CD28 (BD biosciences; San Jose, CA) and recombinant human IL-2 (PeproTech; Rocky Hill, NJ) for 2 days prior to lentivirus infections and for another 3 days post transfection. For anti-PD-1 treatment and IL-2 administration experiment, CD4 T cells isolated from HS were cultured for 24 h in the presence of Dynabeads coated with human T cell activator anti-CD3/CD28, anti-PD-1 (nivolumab, 20 μg/ml; Selleckchem, TX), human IgG4 isotype control antibody antibodies (Biolegend), or IL-2 (100 units/ml; Biolegend).

### Lentiviral-Mediated shRNA Knockdown or Overexpression

The following constructs were used in this study: GreenPuro Scramble Hairpin Control (System Biosciences; Palo Alto, CA), miRZip-21 anti-miR-21 (System Biosciences), and control vector and GAS5 constructs as described previously (24). Gas5-mutant construct was generated using Q5 Site-Directed Mutagenesis Kit (New England BioLab, E0554S) according to manufacturer's protocol. Briefly, GAS5 WT vector was used as the template for site-directed mutagenesis PCR with primers (sequences shown in Table 1) that target exon 4 of GAS5 WT transcript, abolishing the miR-21 binding site on GAS5. The primers were generated using NEBaseChanger site (New England Biolab). DNA plasmid was transfected into Stbl3™ chemically competent *E. Coli* (Introvigen). The transformed cells were cultured on agar plates, positive colonies were selected for plasmid DNA isolation. The plasmid DNA was then subjected for DNA sequencing to confirm the mutation.

The control, GAS5-WT, and GAS5-mutant vectors were transfected into HEK 293T cells (ATCC) together with psPAX2 and PMD2G vectors to generate the respective lentiviruses using the FuGENE® HD Transduction Reagent (System Bioscience). HEK 293T cells were cultured in DMEM media (Fisher Scientific) containing 10% fetal bovine serum, 100 IU/ml penicillin, and 2 mM L-glutamine at 37°C and 5% CO_2_ atmosphere. The supernatants were collected and the virus particles were concentrated using PEG-it Virus Precipitation Solution (System Bioscience) according to the manufacturer's instructions.

Isolated CD4 T cells from HS or PLHIV were stimulated with soluble anti-CD3 and anti-CD28 for 2 days before lentiviral infection. The transdux max (System Bioscience) reagent was used followed the manufacturer protocol to enhance the infection efficiency. CD4 T cells were harvested 3 days post infection and subjected for further experiments.

### RNA Extraction, cDNA Synthesis, and Real-Time RT-PCR

Total RNA was extracted from ~1 × 10^6^ CD4 T cells isolated from each subject using the RNeasy Mini Kit (Qiagen; Germantown, MD). Approximately 100 ng of RNA was reverse transcribed using the High Capacity cDNA Reverse Transcription Kit (Applied Biosystems; Foster City, CA). For miRNA measurements, miRNA was extracted using the miRNeasy Micro kit (Qiagen) and cDNA was synthesized using the TaqMan Advanced miRNA cDNA Synthesis Kit (Qiagen) following the manufacturer's instructions.

Quantitative real-time PCR was performed using iTag Universal SYBR Green Supermix (Bio-Rad; Hercules, CA). Gene expression was determined using the 2^−ΔΔct^ method and normalized to GAPDH (for mRNA and lncRNA expressions) and miR-191-5p (for miRNA expression), and the results are presented as relative fold changes. The PCR primers are listed in Table 2.

**Table 2:** Primer sequences used for RT-qPCR experiments in the paper.

**Primer sequences**	**Supplier**	**Catalog number**
Gapdh forward, GGATTTGGTCGTATTGGG	Thermo Fisher	N/A
Gapdh reverse, GGAAGATGGTGATGGGATT	Thermo Fisher	N/A
Gas5 forward, GGACCGGGAGATAGGAGTG	Thermo Fisher	N/A
Gas5 reverse, CACGGACTCCAGGTGATGAG	Thermo Fisher	N/A
Gas5-mutant forward, ACAGATCAAGGTGAAGTTGAAATGGTGGAGTC	Thermo Fisher	N/A
Gas5-mutant reverse, AATTCATTTGTGTGCCAATGGCTTGAGTT	Thermo Fisher	N/A
hsa-miR-21-5p	Fisher Scientific	477975_A26676
hsa-miR-191-5p	Fisher Scientific	477952_A26676

*This table provides the primer sequences, the supplier information and catalog number of each primer used for RT-qPCR experiments in this study*.

### Flow Cytometry

Sample preparation and surface and intracellular marker staining were performed using the following antibodies, according to the manufacturer's protocols: anti-human CD279 (PD-1)/PE [NAT105] (35), H2A.X Phospho (Ser139)/PE [2F3], CD69/FITC [FN50], IL-2/PE [MQ1-17H12], IFN-γ /PE [4S.B3], TNF-α /APC [MAb11], CD366 (Tim-3)/APC [F38-2E2], Phospho-AKT/ APC (Ser473) [SDRNR], MAPK/Alexa Fluor 488 [pT180/pY182], Bcl-2/Alexa Fluor 647 [C-2], and TGF-β1/PE [TW4-2F8] (all from Biolegend; San Diego, CA). The cells were stimulated with PMA/ionomycin (Sigma-Aldrich; St. Louis, MO) for 4–6 h before the detection of IL-2, IFN-γ, TNF-α, and TGF-β1. For apoptosis analysis, cells were stained with Annexin V and 7-AAD from the PE Annexin V apoptosis detection kit (BD Biosciences; San Jose, CA). Active caspase-3 was detected using a cleaved caspase-3 staining kit (FITC or Red) following the manufacturer's instructions (Abcam; Cambridge, MA). For cell cycle progression, TCR-stimulated CD4 T cells from PLHIV and HS were harvested at days 1, 3, and 5 and stained with Propidium Iodide (PI) using PI flow cytometry kit (Abcam) following the manufacturer's protocol. Samples were acquired on a BD Accuri^TM^ C6 flow cytometer and analyzed using Flowjo software.

### Western Blot

Western Blot was performed as described previously (33). The primary antibodies used were rabbit PARP-1 Ab (clone 46D11; Cat #9532), rabbit phospho-histone H2A.X Ab (Ser139) (clone 20E3; Cat #9718), rabbit phospho-S6 ribosomal protein (Ser235/236) Ab (clone D57.2.2E; Cat #4858), rabbit phospho-Akt (Ser473) XP Ab (clone D9E; Cat #4060), mouse AGO2 Ab (clone 2E12-1C9; Cat #57113), and rabbit GAPDH XP mAb (clone D16H11; Cat #5174) (all from Cell Signaling Technology; Danvers, MA). After washing, the blots were incubated with appropriate horseradish peroxide-conjugated secondary antibodies (Cell Signaling), and protein bands were developed with the Amersham ECL Prime Western Blotting Detection Reagent (GE Healthcare). The chemiluminescent signal was detected and quantified by Chemi Doc^TM^ MP Imaging System (Bio-Rad) and normalized to GAPDH.

### Statistical Analysis

The data were analyzed using Prism 7 software and are presented as mean ± SEM or median with interquartile range. Statistical outliers were removed by the ROUT method (*Q* = 1%). Based on whether the values passed the D'Agostino-Pearson normality or Kolmogorov-Smirnov test, either paired Student's *t*-test or Wilcoxon matched-pairs signed-rank test was used for the analysis involving knockdown/overexpression experiments. Likewise, independent unpaired Student's *t*-test with Welch's correction or Mann Whitney *U*-test for non-paired experiments. Correlations were analyzed by either Pearson's or Spearman's correlation, depending on the data normal distribution test. *P* < 0.05 and *P* < 0.001 were considered statistically significant and very significant, respectively.

## Results

### Differential Regulation of GAS5 and miR-21 Expressions in CD4 T Cells Derived From PLHIV and HS

**Figure 1:**
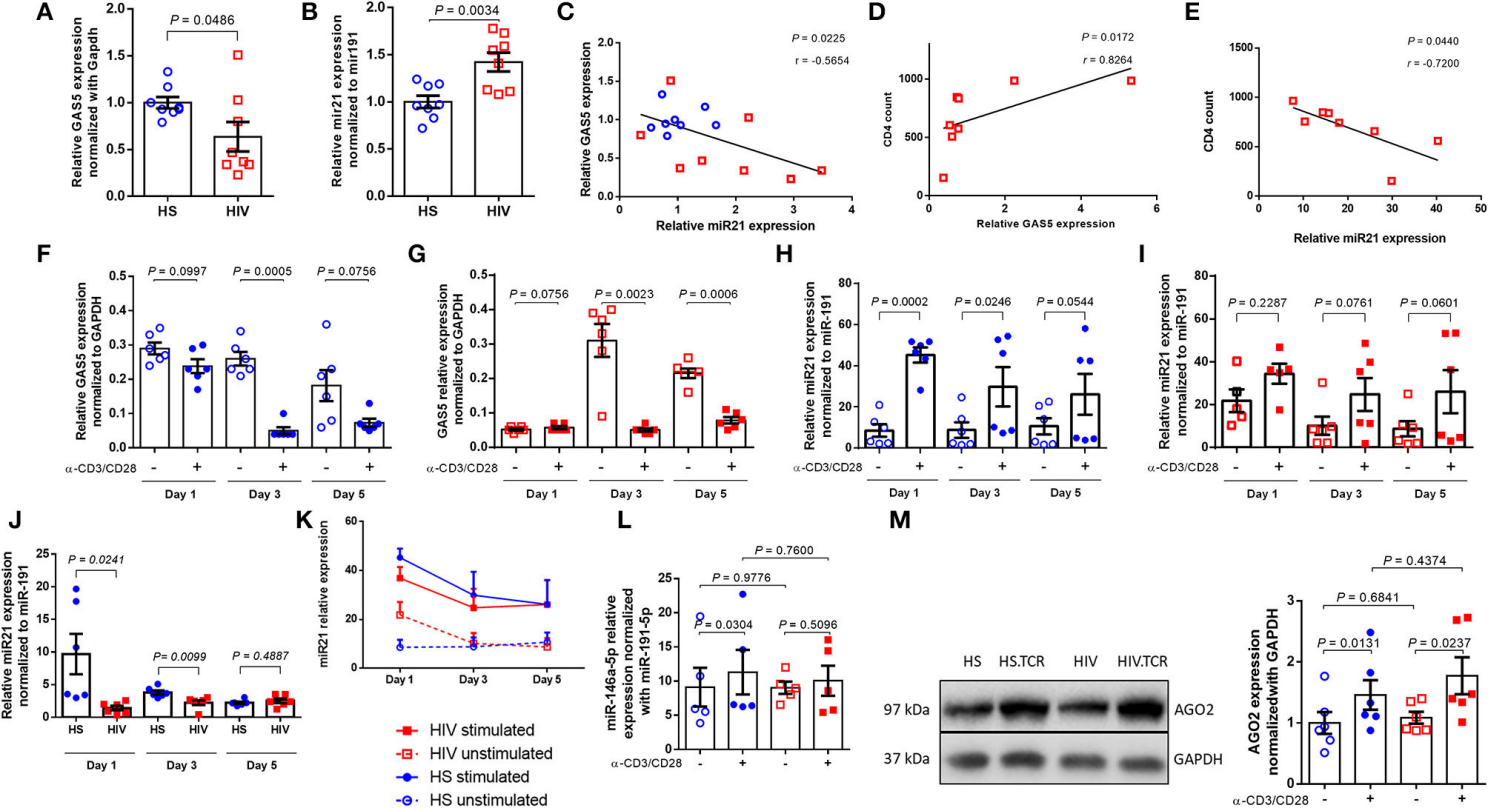
Differential expression of GAS5 and miR-21 in CD4 T cells after TCR stimulation. **(A,B)** Relative expression of GAS5 (*n* = 8) and miR-21 (*n* = 8) in CD4 T cells isolated from HS and PLHIV was determined by RT-qPCR. **(C)** Correlation between GAS5 and miR-21 expressions in CD4 T cells isolated from HS or PLHIV was determined by Pearson correlation analysis. **(D,E)** Correlation between CD4 T cell counts and relative GAS5 (*n* = 8) or miR-21 (*n* = 8) expressions in CD4 T cells from PLHIV was determined by Pearson Correlation analysis. **(F,G)** Relative expression of GAS5 in CD4 T cells from HS and PLHIV in the absence or presence of TCR stimulation for 1, 3, and 5 days (*n* = 6). **(H,I)** Relative expression of miR-21 in HS and PLHIV CD4 T cells with or without TCR stimulation for 1, 3, and 5 days (*n* = 6). **(J)** Relative expression of miR-21 in TCR-stimulated CD4 T cells from HS and PLHIV in 1, 3, and 5 days (*n* = 6). **(K)** Kinetic changes in miR-21 expression in CD4 T cells from both PLHIV and HS following TCR stimulation for 1, 3, and 5 days. **(L)** Relative expression of miR-146a in CD4 T cells from HS and PLHIV with or without TCR stimulation for 24 h (*n* = 6). **(M)** Representative dot blot and relative expression of AGO2 (a major component of RISK complex) in CD4 T cells from HS and PLHIV with or without TCR stimulation for 24 h, determined by Western blotting (*n* = 6).

While thousands of lncRNAs have been identified in human cells, only a small number have been experimentally validated and shown to be associated with human diseases, particularly with infectious diseases. Among these lncRNAs, GAS5 has been shown to simultaneously target multiple genes involved in cell growth and apoptosis. As a first step to elucidate the role of GAS5 in T cell regulation during HIV infection, we measured its expression in CD4 T cells isolated from ART-controlled PLHIV and age-matched HS. Because human GAS5 encodes several splicing isoforms (GAS5a and GAS5b, in addition to 5 GAS5 EST sequences) (37), we synthesized a primer set within exon 12, which can amplify all known isoforms of GAS5, and measured expression levels by real-time RT-PCR. As shown in Figure 1A, GAS5 transcripts were significantly lower in CD4 T cells from PLHIV compared with HS (*n* = 8 subjects).

In addition to targeting protein-coding genes, lncRNAs can also target miRNAs to regulate cellular responses and tumor progression. Bioinformatics analysis using the RNA22 program revealed that GAS5 has sequences complementary to several miRNAs, including miR-21. We were interested in miR-21 because its expression is dynamically upregulated and regulates T cell responses during T cell activation and differentiation. To determine whether GAS5 plays a role in T cell dysfunctions via regulating miR-21 expression during HIV infection, we measured miR-21 levels in CD4 T cells isolated from ART-controlled PLHIV and HS by RT-qPCR. As shown in Figure 1B, miR-21 expression was significantly increased in CD4 T cells derived from PLHIV compared with HS (*n* = 8). Since miR-21 is aberrantly upregulated during T cell activation and differentiation, these results are in line with the overall immune activation driven by chronic inflammation in ART-controlled, latent HIV infection. Next, we compared miR-21 and GAS5 expressions in T cells derived from the same subjects. As shown in Figure 1C, the decline in GAS5 levels closely correlated with miR-21 upregulation in CD4 T cells from the same HIV patients and HS (*n* = 8 subjects). Also, the level of GAS5 positively correlated, whereas miR-21 negatively correlated, with the CD4 T cell counts in the peripheral blood of ART-controlled PLHIV (Figures 1D,E). Taken together, these results indicate that GAS5 is downregulated, whereas miR-21 is upregulated in CD4 T cells during HIV infection, and that their expressions correlate with CD4 T cell counts in the peripheral blood of ART-controlled PLHIV.

### Differential Expression of GAS5 and miR-21 Following TCR Stimulation of CD4 T Cells From PLHIV and HS

Although ART controls viral replication, PLHIV still exhibit low-grade inflammation and immune activation, driving T cell exhaustion and senescence. We thus hypothesized that the differential expression of GAS5 and miR-21 in CD4 T cells is likely due to overall immune activation, as additional TCR stimulation *in vitro* could drive aberrant RNA expressions further, but the response of CD4 T cells from PLHIV may be attenuated due to their exhausted and senescent status. To test this hypothesis, we cultured CD4 T cells from PLHIV and age-matched HS (*n* = 6) with or without Dynabeads-coated with anti-CD3 and anti-CD28 antibodies for 1, 3, and 5 days and measured GAS5 transcripts by RT-qPCR. As shown in Figure 1F, TCR stimulation decreased GAS5 levels in CD4 T cells from HS at day 1 (*p* = 0.0997), day 3 (*p* = 0.0005), and day 5 (*p* = 0.0756), but such a decrease was only statistically significant at day 3 compared to the unstimulated cells. Similarly, GAS5 levels in CD4 T cells from PLHIV were also significantly downregulated following TCR stimulation at day 3 (*p* = 0.0023) and day 5 (*p* = 0.0006), but not at day 1 (*p* = 0.0756; Figure 1G). These results demonstrate that TCR stimulation reduces GAS5 expression in CD4 T cells derived from both HS and PLHIV.

To determine the effects of T cell activation on miR-21 regulation, we also measured miR-21 levels in CD4 T cells from the same group of PLHIV and age-matched HS (*n* = 6 subjects) after TCR stimulation for 1, 3, and 5 days. Following stimulation with bead-conjugated anti-CD3 and anti-CD28 antibodies, HS CD4 T cells responded robustly and kinetically, with an increasing miR-21 expression, particularly at day 1 (>5-fold increase, *p* = 0.0002) and day 3 (>3-fold increase, *p* = 0.0246; Figure 1H). Although HIV CD4 T cells also exhibited increases in miR-21 expression after TCR stimulation, the differences between unstimulated and stimulated cells were not significant (fold-increases between 1 and 2 overtime, all *p* > 0.05; Figure 1I). These results indicate that CD4 T cells from PLHIV respond to TCR stimulation poorly compared to those from HS. Notably, miR-21 levels were remarkably higher in CD4 T cells from PLHIV than HS without TCR stimulation *in vitro* (Figure 1B), and were significantly lower in TCR-stimulated CD4 T cells from PLHIV after the values were normalized to the unstimulated cells, especially at day 1 (6.85-fold decrease, *p* = 0.0241) and day 3 (1.7-fold decrease, *p* = 0.0099). The day 5 stimulation resulted in a slightly higher miR21 expression in HIV samples (1.13-fold increase, *p* = 0.4887; Figure 1J). Also, the changes in miR-21 expressions in CD4 T cells from both PLHIV and HS following TCR stimulation *in vitro* for 1, 3, and 5 days are shown in Figure 1K and clearly show that miR-21 was upregulated in CD4 T cells from both PLHIV and HS after TCR stimulation. However, the response of CD4 T cells from PLHIV was attenuated compared to cells from HS, especially at the early phase (day 1–3) of TCR stimulation. Since the day 0 (baseline) data for GAS5 and miR-21 expressions without TCR stimulation are shown in Figures 1A,B, we did not include this time point when we set up the TCR stimulation for the kinetic experiments due to limited availability of the cells, which we recognize as a limitation for the analysis of these data (Figures 1F–K).

Previous studies have shown that miR-146a is upregulated in TCR-stimulated T cells and in HIV-1-infected individuals (38). Also, the expression of miR-146 was found to be correlated with the expressions of T cell exhaustion and senescence markers, such as PD-1, Tim-3, and LAG-3 (38). Our data in Figure 1L show that the levels of miR-146a were increased in CD4 T cells from HS (*p* = 0.0304), but not PLHIV (*p* = 0.5096), following TCR stimulation for 24 h. Also, we found no significant differences in miR146 levels in CD4 T cells derived from PLHIV and HS, in both unstimulated (*P* = 0.9776) and TCR-stimulated (*P* = 0.7600) conditions (Figure 1L).

Argonaute 2 (AGO2) is the catalytic component of RISC that recognizes and cleaves mRNA of complementary sequences. From a duplex, one strand is preferentially loaded into AGO2 to generate a functional miRNA-induced silencing complex (miRISC). We asked whether levels of AGO2 are affected during TCR stimulation. To address it, we measured AGO2 in CD4 T cells from HS and PHLIV with and without TCR stimulation for 24 hours. Although AGO2 expression was increased after TCR stimulation in CD4 T cells from both HS and PHLIV sequence, there were no significant differences in AGO2 levels, between the two groups, in both unstimulated and stimulated (Figure 1M). These data indicate that AGO2 expression levels are significantly increased, with no differences between HS and HIV suggesting that TCR stimulation may affect widely miRISC activity.

### Effects of IL-2 and Anti-PD1 Treatment on the GAS5-miR-21-Axis

**Figure 2:**
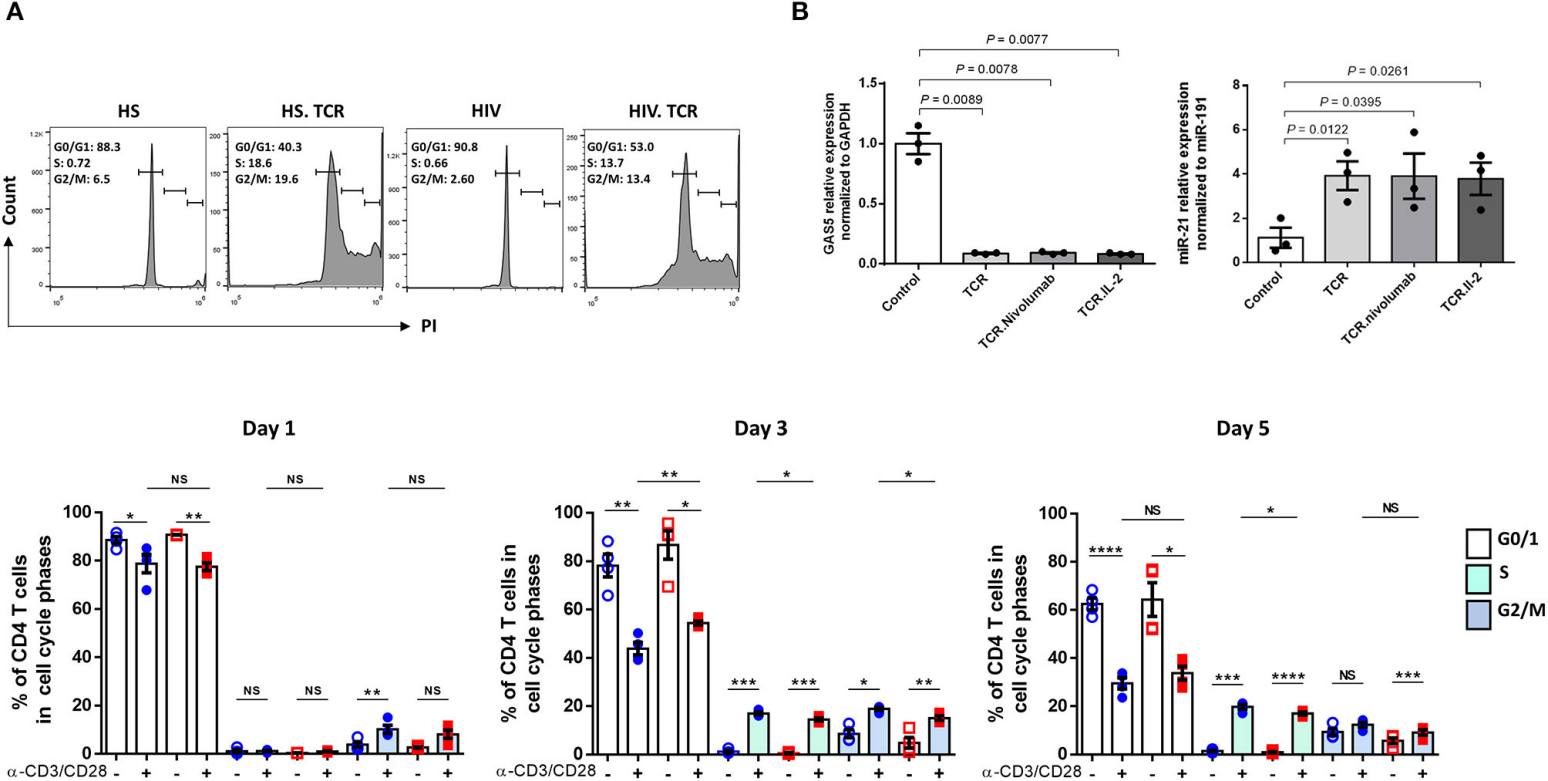
Cell cycle progression, AGO2 expression, and effect of anti-PD1/IL-2 in TCR-stimulated CD4 T cells on GAS5-miR-21 axis. **(A)** Cell cycle progression in CD4 T cells from HS and PLHIV following TCR stimulation at day 1, 3, and 5 measured by PI flow cytometric analysis (*n* = 4). Representative histogram of cell cycle phases of CD4 T cells from HS and PLHIV with and without TCR stimulation at day 3 are shown above and summary data at day 1, 3, 5 are shown below. **(B)** Relative expression of GAS5 and miR-21 in CD4 T cells from HS and PLHIV following anti-PD1 and IL-2 treatments for 24 h, determined by RT-qPCR (*n* = 3). n.s, *P* > 0.05; *P ≤ 0.05; **P ≤ 0.01; ***P ≤ 0.001; ****P ≤ 0.0001.

To further define the effect of GAS5-miR-21 on T cell activities, we measured cell cycle progression by PI staining in CD4 T cells from HS and PLHIV, following TCR stimulation for 1, 3, and 5 days. As shown in Figure 2A (representative histogram for numbers of CD4 T cells in G0/1, S, and G2/M phases at day 3 and summary data at day 1, 3, and 5), there were significant differences in the percentages of CD4 T cells present in S and G2/M phases of cell cycle with and without TCR stimulation. Notably, the percentages of CD4 T cells from HS and PLHIV in various cell cycle phases were comparable after TCR stimulation for 1 day. We found a significant higher percentage of CD4 T cells from HS were going through cell cycle phases compared to those from PLHIV following TCR stimulation for 3–5 days, particularly at day 3. These data indicate that TCR stimulation induces cellular proliferation and division, but as the expression GAS5 and miR-21 is altered in response to TCR stimulation, CD4 T cells from PLHIV behave differently from HS; in that HS-CD4 T cells respond relatively stronger and ultimately progress to T cell exhaustion and senescence with persistent stimulation.

Attempts have been made to restore dampened T cell functions in PLHIV via manipulating some of the dysregulated signaling pathways. For instance, blockade of the inhibitory PD-1/PD-L1 pathway enhanced viral-specific CD4 T cell response along with a decreased viral replication (39, 40). In addition, administration of IL-2 led to CD4 T cell expansion, and such expansion was coupled with a decreased turnover and improved survival of naive and central memory CD4 T cells, leading to recovery of T cell function and immune response (41, 42). Here, we examined whether PD-1 blockade with IL-2 signaling can impact the GAS5/miR-21 axis during TCR stimulation by determining the levels of GAS5 and miR-21 in TCR-stimulated HIV-CD4 T cells in the presence of PD-1 inhibitor (Nivolumab) and IL-2. As shown in Figure 2B, anti-PD1 and IL-2 treatment could not restore the aberrant GAS5/miR-21 expressions following TCR stimulation in CD4 T cells from PLHIV.

### PLHIV on ART Exhibit T Cell Dysregulation and Apoptosis due to Aberrant DDR

**Figure 3:**
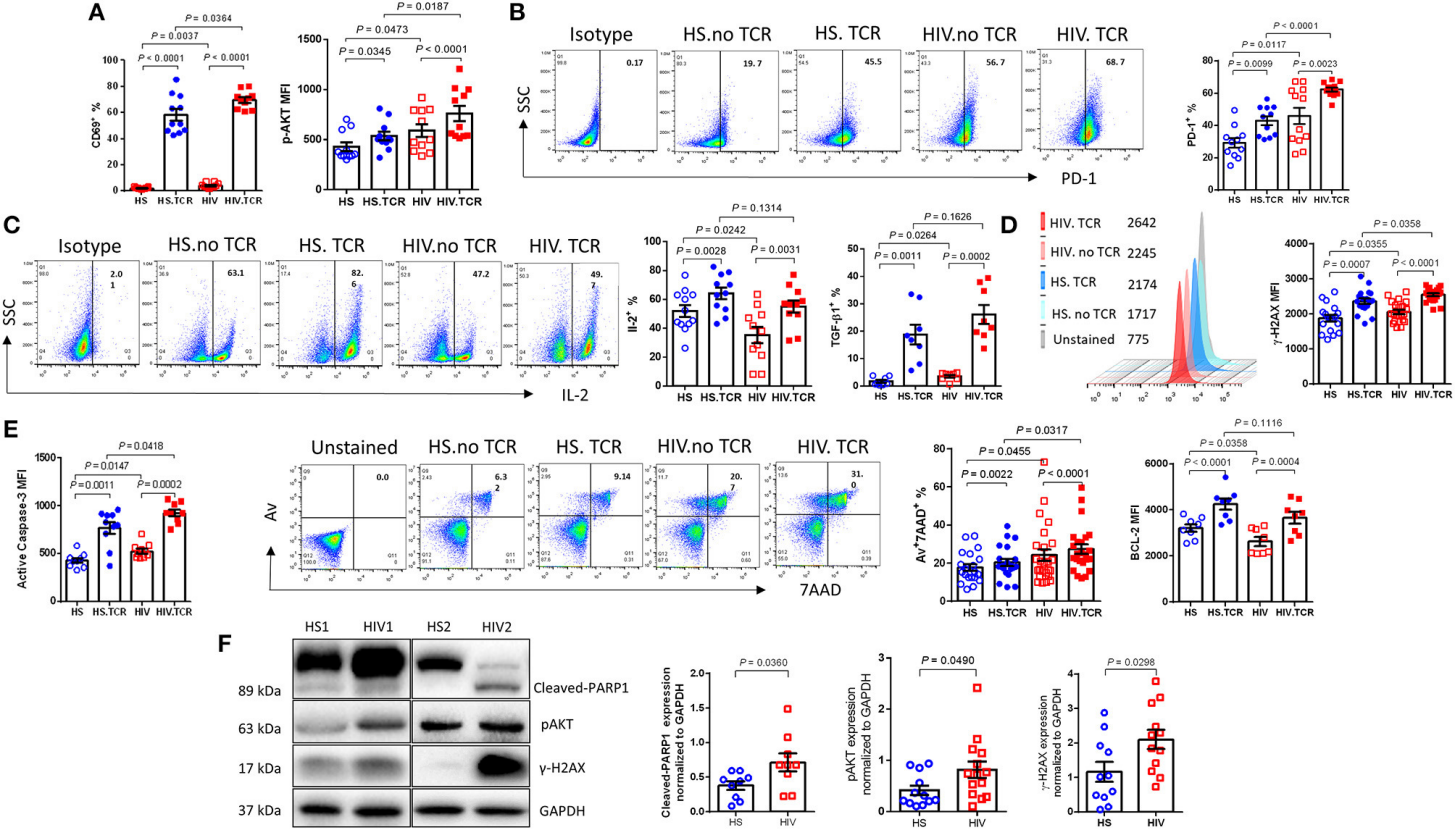
PLHIV on ART exhibit CD4 T cell dysregulation and apoptosis due to aberrant DDR. T cell activation, exhaustion, cytokine production, apoptosis, and DNA damage marker expressions in TCR-stimulated or non-stimulated CD4 T cells isolated from HS or PLHIV were determined by Flow Cytometry and Western Blot. **(A)** Frequency of CD69^+^ (*n* = 11) and MFI of pAKT^+^ cells (*n* = 11). **(B)** Representative dot plots and summary data of PD-1^+^ cells (*n* = 12). **(C)** Representative dot plots and summary data of IL-2 expression (*n* = 12), expression of TGFβ1 (*n* = 8). **(D)** Representative histogram and summary MFI of γH2AX in CD4 T cells from HS (*n* = 18) and PLHIV (*n* = 21). **(E)** Expression of active caspase-3 (*n* = 10), representative dot plots and summary data of Av^+^7AAD^+^ CD4 T cells from HS (*n* = 21) and PLHIV (*n* = 25), expression of BCL-2 in CD4 T cells from HS and PLHIV (*n* = 8). **(F)** Representative imaging and summary data of immunoblotting for PARP-1, pAKT, and γH2AX, normalized by GAPDH.

To determine the possible effects of GAS5/miR-21 on the cellular response during latent HIV infection, we examined the expressions of known protein markers for T cell activation, exhaustion, DNA damage, cellular functions, and apoptosis using flow cytometry and western blotting. As shown in Figure 3A, the frequencies of CD69 (an early activation marker) positive cells were significantly increased upon TCR stimulation of CD4 T cells from both PLHIV and HS; however, PLHIV exhibited significantly increased T cell activation at baseline prior to stimulation. Correspondingly, phosphorylation of AKT (pAKT) was upregulated in CD4 T cells from PLHIV after TCR stimulation. Because CD4 T cells are persistently activated during HIV infection, they expressed higher levels of the activation and exhaustion marker programmed death-1 (PD-1) before and after TCR stimulation compared to CD4 T cells from HS (Figure 3B). Likewise, CD4 T cells from PLHIV showed relatively lower levels of the survival cytokine interleukine-2 (IL-2) (Figure 3C), whereas levels of the pro-inflammatory cytokine TGF-β1 were upregulated after TCR stimulation compared to CD4 T cells from HS.

We have recently reported that chronic inflammation in ART-controlled PLHIV induces telomeric DNA damage and cell apoptosis. Here, we found that phosphorylation of the H2A histone family member γH2AX (a marker of DNA damage) was significantly upregulated by TCR stimulation in CD4 T cells derived from both PLHIV and HS, and was further increased in T cells from PLHIV, with or without TCR stimulation (Figure 3D). Correspondingly, PLHIV had a high level of CD4 T cell apoptosis, as evidenced by the significant increase in cleaved caspase-3 and Av/7AAD levels, perhaps due to reduced levels of Bcl-2 expression (an anti-apoptotic molecule), in CD4 T cells under both unstimulated and stimulated conditions compared to HS (Figure 3E). The DNA damage-mediated cell apoptosis was confirmed by western blot, which showed increases in the levels of cleaved PARP-1, pAKT, and γH2AX in CD4 T cells from PLHIV compared to HS (Figure 3F). These results suggest that CD4 T cells from PLHIV exhibit a phenotype of over-activation, exhaustion, DNA damage, and cell apoptosis.

### miR-21 Controls CD4 T Cell Response and Apoptosis via Regulating the TCR Signaling Pathways in PLHIV

**Figure 4:**
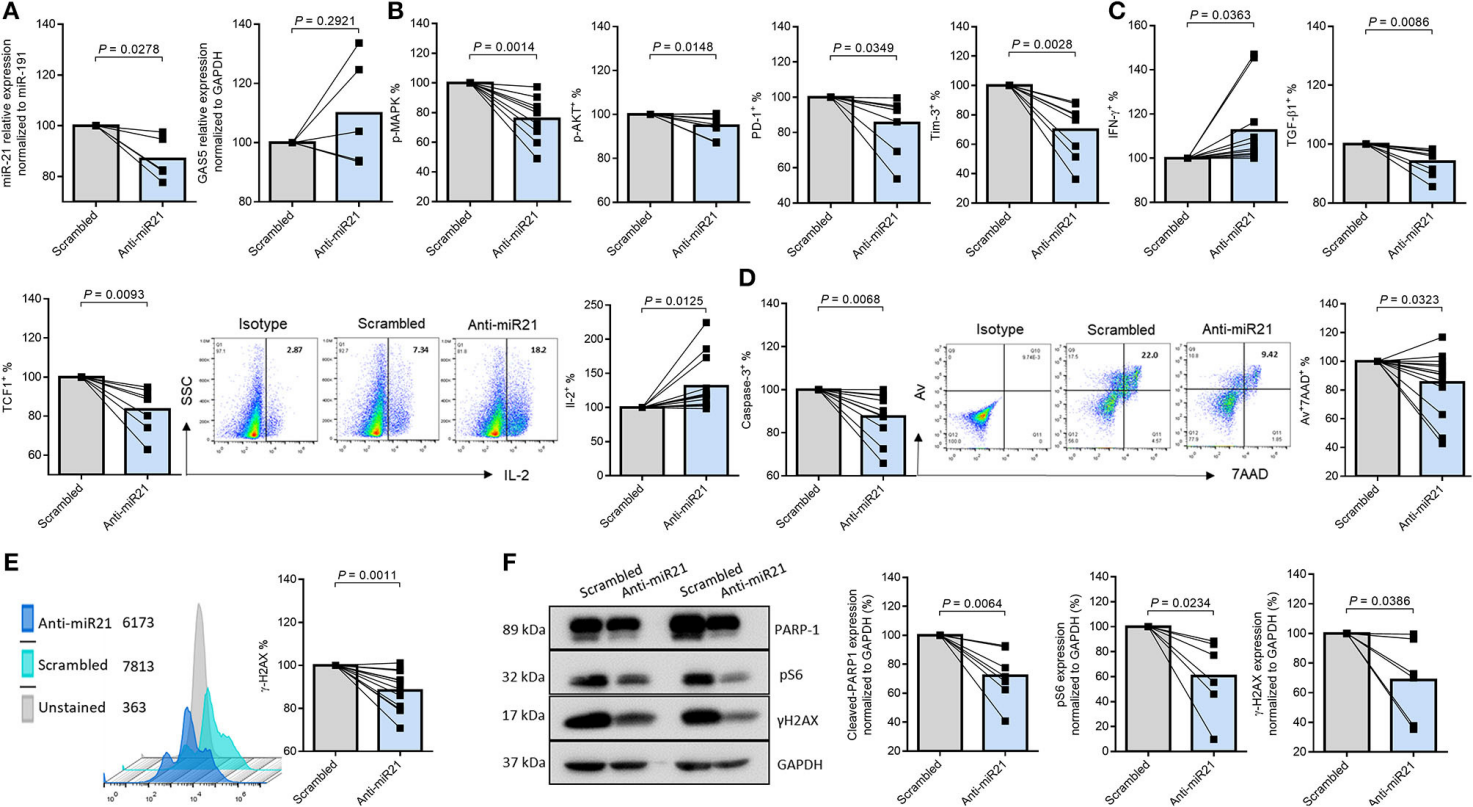
MiR-21 controls CD4 T cell response and apoptosis via regulating the TCR signaling pathways in PLHIV. T cell activation, exhaustion, cytokine production, apoptosis, and DNA damage marker expressions after miR-21 knockdown in CD4 T cells from PLHIV were determined by Flow Cytometry and Western Blot. **(A)** Relative expression of miR-21 and GAS5 following miR-21 knockdown in CD4 T cells from PLHIV determined by RT-qPCR (*n* = 5). **(B)** Frequencies of pMAPK (*n* = 9), pAKT (*n* = 9), PD-1 (*n* = 11), and Tim-3 (*n* = 8). **(C)** Representative dot plot and summary data of IL-2 (*n* = 16), IFN-γ (*n* = 16), TGFβ1 (*n* = 8), and TCF1 (*n* = 7) expressions. **(D)** Representative dot plot and summary data of Av^+^ 7AAD^+^ CD4 T cells (*n* = 13), expression of active caspase-3 (*n* = 10). **(E)** Representative histogram and summary data of γH2AX expression (*n* = 12). **(F)** Representative western blots and summary data of cleaved PARP-1 (*n* = 8), pS6 (*n* = 6), and γH2AX (*n* = 6) expressions, normalized to GAPDH.

To examine whether and how miR-21 controls T cell response during HIV infection, we transfected CD4 T cells with lentiviral shRNA against miR-21, and assessed the markers for T cell activation, exhaustion, DNA damage, and cellular apoptosis by flow cytometry and western blotting. Figure 4A shows knockdown of miR-21 as determined by a slight decrease in its levels in CD4 T cells transfected with anti-miR21 compared to the control vector. Notably, knockdown of miR-21 did not change the expression of GAS5, indicating that GAS5 expression is not regulated by miR-21. As shown in Figure 4B, the frequencies of pAKT^+^, pMAPK^+^, PD-1^+^, and Tim-3^+^ cells were significantly reduced after miR-21 knockdown. Additionally, IL-2 and IFN-γ expressions were increased, whereas the number of TGF-β1 expressing CD4 T cells from PLHIV was significantly decreased (Figure 4C), after miR-21 knockdown.

It has been reported that T cell factor-1 (TCF1) critically regulates T cell activation and differentiation through activation of the canonical Wnt signaling pathway during TCR stimulation (43). We thus examined TCF1 expression in PLHIV CD4 T cells following miR-21 knockdown. As shown in Figure 4C, the percentage (%) of TCF1^+^ cells was significantly decreased in PLHIV CD4 T cells transfected with anti-miR-21 siRNA compared to the control. Moreover, the frequencies of Av^+^/7AAD^+^ cells and the MFI of cleaved caspase-3 (Figure 4D) and the MFI of DNA damage marker γH2AX (Figure 4E) were significantly decreased, in PLHIV CD4 T cells with miR-21 knockdown. To further confirm the changes in the expression of these markers, we measured their levels by Western blotting. As shown in Figure 4F (representative blots and summary data), the levels of cleaved PARP-1, pS6, and γ-H2AX were downregulated after miR-21 knockdown. These results indicate that disrupting miR-21-mediated signaling can rescue PLHIV CD4 T cells from over-activation, exhaustion, cytokine inhibition, DNA damage, and cell apoptosis.

### GAS5 Negatively Regulates miR-21 Expression in TCR-Activated CD4 T Cells From PLHIV and HS

**Figure 5:**
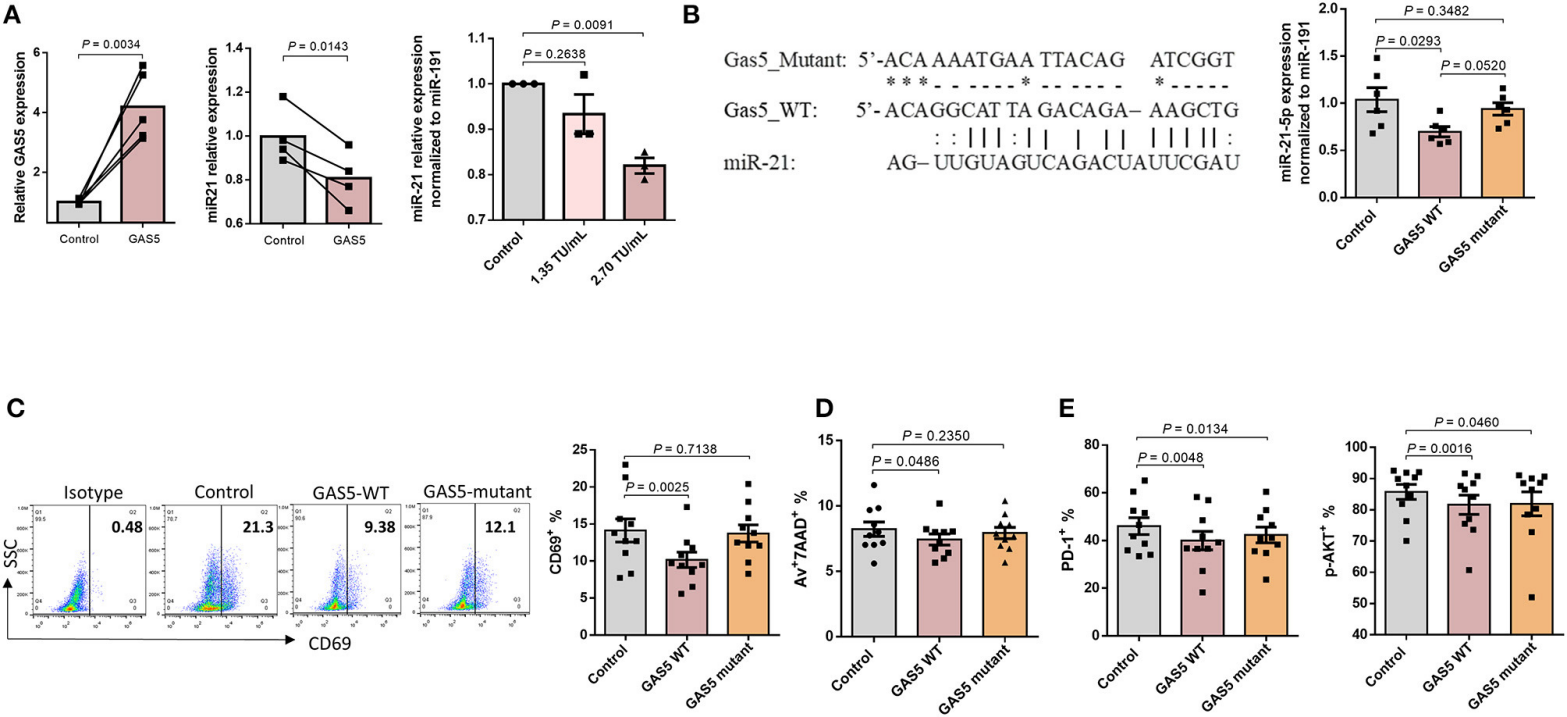
GAS5 controls CD4 T cell response and apoptosis beyond miR-21-mediated signaling pathways. **(A)** Relative expressions of GAS5 (*n* = 5) and miR-21 (*n* = 4) in CD4 T cells from PLHIV, following overexpression of GAS5, determined by RT-qPCR. **(B)** Left panel, sequences of GAS5-mutant and GAS5-WT constructs, and the corresponding miR-21 binding site. Right panel, relative miR-21 expression in CD4 T cells from PLHIV transfected with control, GAS5-WT, or GAS5-mutant constructs (*n* = 6). **(C–E)** Expression of T cell activation, apoptosis, and exhaustion markers in CD4 T cells from PLHIV transfected with control, GAS5-WT, or GAS5-mutant construct, determined by flow cytometry (*n* = 10).

How GAS5 and miR-21 are differentially regulated by TCR stimulation is poorly understood. As stress-inducible genes, GAS5 and miR-21 are induced in response to cellular stress conditions, such as serum starvation or TCR stimulation. Although previous studies have shown that GAS5 and miR-21 regulate each other's expression in a mutually exclusive way, our results in Figure 4A showed that GAS5 expression was not significantly increased after miR-21 knockdown in PLHIV CD4 T cells. In addition, we determined the effect of GAS5 on miR21 expression by overexpressing GAS5 in TCR-activated PLHIV CD4 T cells using the lentivirus expression system, followed by examining GAS5 and miR21 expression. The data in Figure 5A confirmed a significant increase in GAS5 levels in TCR-stimulated HIV-CD4 T cells (2 days) transfected with GAS5 (*p* = 0.0034) for additional 3 days. We next determined the levels of miR-21 in CD4 T cells overexpressing GAS5. As shown in Figure 5A, the level of miR-21 was downregulated in a dose-dependent manner, depending on the amount of the lentivirus (0, 1.35 × 10^6^ TU/mL, and 2.70 × 10^6^ TU/mL) to overexpress GAS5, indicating that miR-21 level is regulated by GAS5 in TCR-activated PLHIV CD4 T cells. These results demonstrate that GAS5 negatively regulates miR-21 in TCR-activated PLHIV CD4 T cells and provide a further evidence of an endogenous competitive RNA regulatory network.

### GAS5 Controls CD4 T Cell Responses and Apoptosis During HIV Infection by Regulating Pathways Beyond miR-21-Mediated Signaling

GAS5 not only regulates miR-21 expression, but also other miRNAs involved in cellular differentiation and proliferation (44, 45). To investigate the regulatory effect of GAS5 on CD4 T cells during HIV infection, independently from miR-21-mediated signaling, we generated a GAS5 mutant where amino acids at exon 4 of GAS5 transcript were altered (GAS5-mutant), as depicted in Figure 5B. This mutation abolishes the binding site of miR-21 on GAS5 and thus restricts the interaction between GAS5 and miR-21. Notably, transfection with GAS5-mutant did not significantly alter miR-21 levels compared to control vector, whereas transfection with GAS5-WT significantly decreased miR-21 level (*p* = 0.0293), suggesting that overexpression of GAS5-mutant with no binding affinity for miR-21 does not affect miR-21 expression. We next determined the effect of overexpressing GAS5-mutant on T cell activation and responses. As shown in Figure 5C, the expression of early T cell activation marker CD69, was significantly (*p* = 0.0025) downregulated in CD4 T cells transfected with GAS5-WT compared to the control vector transfection. However, the CD69 expression was partially restored in CD4 T cells overexpressing GAS5-mutant compared to the GAS5-WT, and this change was not statistically significant compared to the control. In addition, we observed a significantly lower apoptosis rate in CD4 cells overexpressing GAS5-WT, but not GAS5-mutant compared, to the control vector (Figure 5D). Nevertheless, the frequencies of T cells expressing PD-1 and pAKT were significantly downregulated in CD4 T cells overexpressing GAS5-WT and GAS5-mutant compared to the control vector (Figure 5E). These results suggest that while GAS5 regulates HIV-CD4 T cell early activation and apoptosis through miR-21-mediated signaling, other signaling pathways may also be involved in the regulation of T cell activation and exhaustion pathways during HIV infection.

### GAS5 Controls CD4 T Cell Response and Apoptosis by Regulating miR-21-Mediated Signaling During HIV Infection

**Figure 6:**
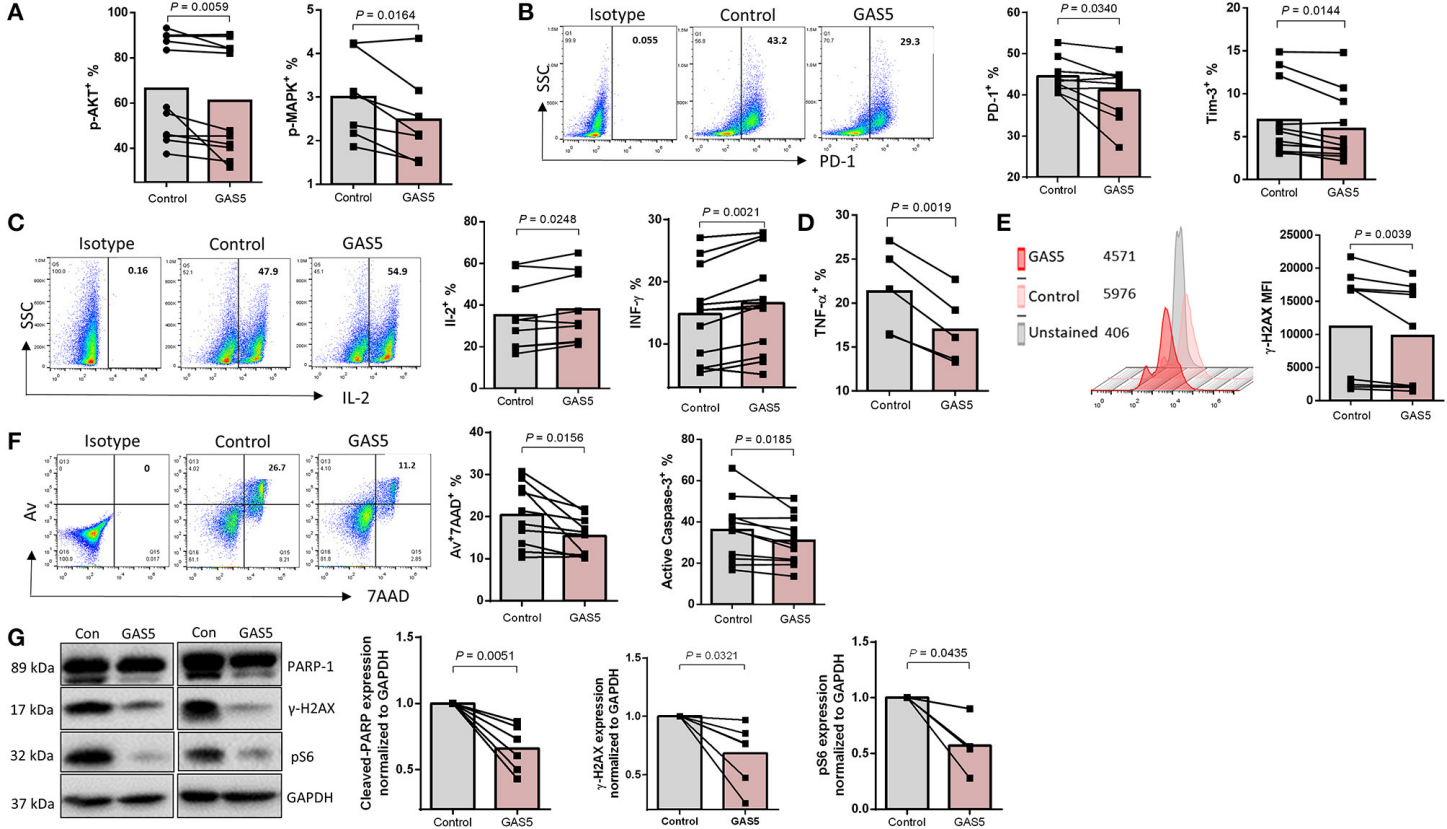
GAS5 controls CD4 T cell response and apoptosis via regulating miR-21-mediated signaling in PLHIV. **(A,B)** Frequencies of pAKT (*n* = 11), pMAPK (*n* = 7), PD-1 (*n* = 10), and Tim-3 (*n* = 11) in control vector or GAS5-transducted CD4 T cells from PLHIV, determined by flow cytometry. **(C,D)** Representative dot plot and summary data of IL-2 expression (*n* = 10), IFN-γ (*n* = 12), and TNF-α (*n* = 5), measured by flow cytometry. **(E)** Representative histogram and summary data of γH2AX expression (*n* = 9). **(F)** Representative dot plot and summary data of Av^+^7AAD^+^ CD4 T cells (*n* = 10) and active caspase-3 (*n* = 11). **(G)** Representative western blot imaging and summary data of cleaved PARP-1 (*n* = 6), pS6 (*n* = 4), and γH2AX (*n* = 6) expressions, normalized to GAPDH.

Having demonstrated a negative regulatory effect of GAS5 on miR-21 expression, we next examined the functional role of GAS5 in CD4 T cell regulation. We investigated whether overexpression of GAS5 has any effect on miR-21-mediated functional changes in CD4 T cells from PLHIV. GAS5 was overexpressed using the lentiviral expression system, and the markers for functional T cells were assessed by flow cytometry and western blotting. As shown in Figure 6A, the frequencies of pAKT^+^ and pMAPK^+^ cells were significantly decreased; PLHIV CD4 T cell exhaustion was reduced, as demonstrated by the downregulation of PD-1^+^ and Tim-3^+^ cell frequencies in GAS5-overexpressing CD4 T cells (Figure 6B). Additionally, IL-2 and IFN-γ producing CD4 T cells were increased (Figure 6C), whereas pro-inflammatory TNF-α producing cells were decreased due to GAS5 overexpression (Figure 6D). Moreover, the level of γH2AX was reduced (Figure 6E), and the cell apoptosis markers Av/7AAD and cleaved caspase-3 were significantly decreased in GAS5 overexpressing CD4 T cells compared to the control vector expression (Figure 6F). To further confirm the changes in the expressions of these markers, we determined their levels by western blotting. As shown in Figure 6G (representative blots and summary data), protein levels of cleaved PARP-1, pS6, and γH2AX, were downregulated in GAS5-overexpressing cells compared to the control vector. These results suggest that increasing GAS5 levels in PLHIV CD4 T cells can negatively regulate miR-21 expression, which in turn improves TCR activation, T cell exhaustion, cytokine production, and reduces DNA damage and cell apoptosis.

## Discussion

**Figure 7:**
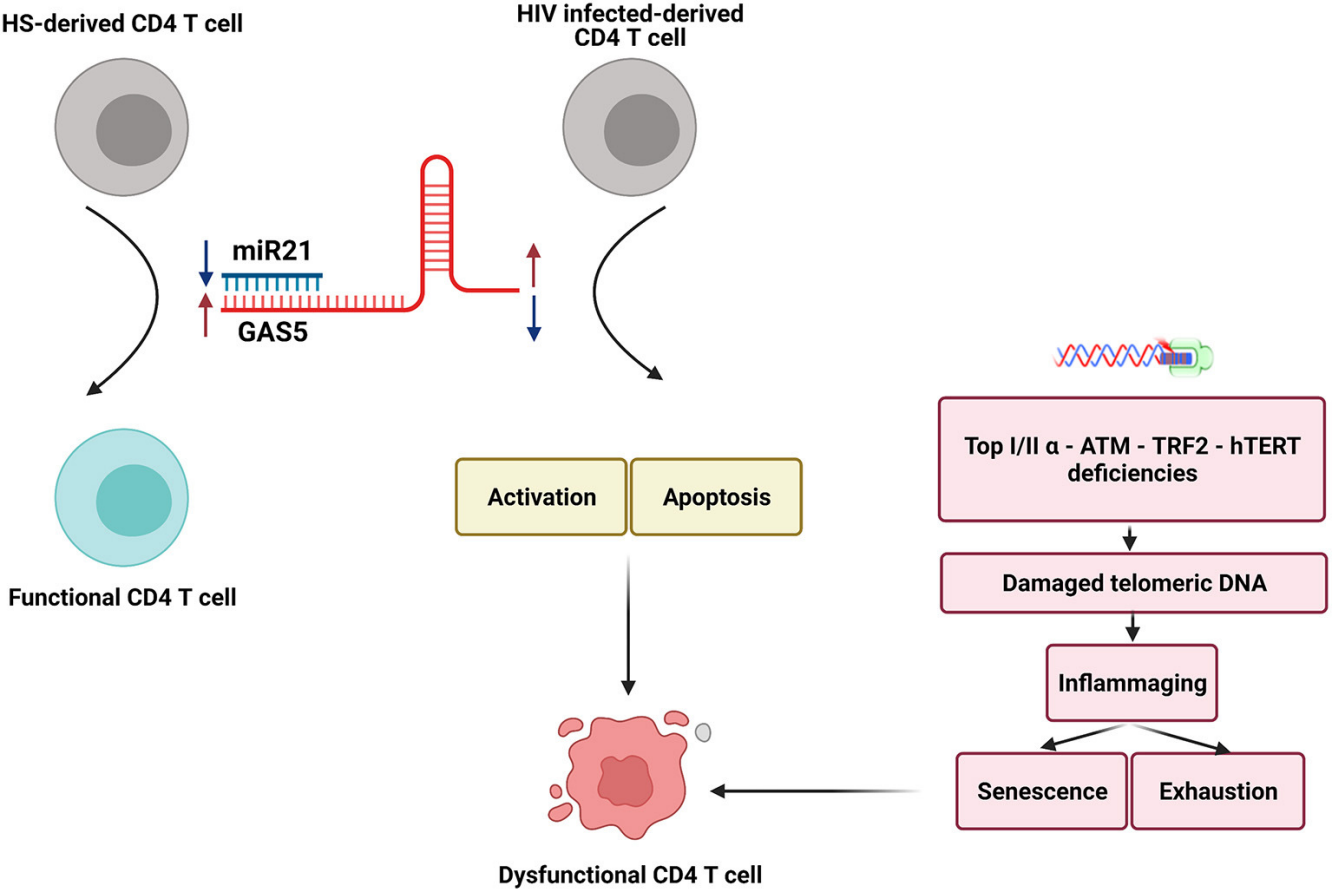
A model depicting how the GAS5-miR-21 axis controls the function and fate of CD4 T cells in PLHIV. Two crucial non-coding RNAs, GAS5 and miR-21 are differentially expressed in CD4 T cells in PLHIV. The decline in GAS5 and increase in miR-21 expressions, along with the deficiencies in crucial enzymatic proteins involved in DNA damage and repair machineries, can cause significant increases in CD4 T cell activation, senescence, apoptosis, DNA damages, and dysregulation of cytokine productions established during latent HIV infection. Importantly, interrupting this GAS5-miR-21 axis may restore T cell homeostasis and competency and resolve premature T cell aging or immune senescence. Notably, GAS5 effects on T cell exhaustion during HIV infection may be mediated by mechanism(s) beyond GAS5-miR21 mediated signaling. Bottom: Deficiencies in Top1/2a, ATM, hTERT, and TRF2 that can cause telomeric DNA damage (30–34, 36) are known to contribute to dysfunctional CD4 T cells in HIV patients.

T cells play an essential role in the control of viral infections. However, T cells in PLHIV–despite successful control of viral replication by ART–are aberrantly dysregulated and exhibit an immune aging phenotype, characterized by CD4 T cell over-activation, exhaustion, senescence, accumulated DNA damage, more apoptosis, and impaired cellular functions. While the causes for this immune aging phenotype have been extensively investigated, the molecular mechanisms underlying these cellular alterations remain incompletely understood. In this study, we demonstrated that GAS5 and miR21 are differentially expressed in CD4 T cells derived from ART-controlled PLHIV and that GAS5 controls miR21 expression to regulate signaling molecules involved in DNA damage and cellular responses following T cell receptor (TCR) stimulation. Based on these new findings and our previous studies–showing deficiencies in topoisomerase I/IIα (Top1/2α), ataxia-telangiectasia mutated, human telomerase reverse transcriptase (hTERT), and telomeric repeat-binding factor 2 (TRF2) that can cause telomeric DNA damage–we propose a model (depicted in Figure 7) illustrating how the GAS5-miR21 axis controls the function and fate of CD4 T cells in PLHIV. Despite the differential expressions of GAS5 and miR-21, the mechanism of miR21-mediated dysregulation of CD4 T cell functions in PLHIV remain unknown. Nevertheless, our data clearly show that the GAS5-miR21 axis can control multiple signaling pathways involved in cell activation, senescence, and apoptosis in PLHIV CD4 T cells. Notably, the decline in GAS5 and the increase in miR21 are associated with the frequencies of CD4 T cells in the peripheral blood of PLHIV. Importantly, GAS5 overexpression or miR21 silencing significantly restored the aberrantly dysregulated T cell activation, DNA damage, apoptosis, and functions of CD4 T cells derived from PLHIV. Additionally, disrupting miR21 binding site in GAS5 transcripts partially restored the GAS5-mediated regulation of CD4 T cell activation and apoptosis but not cell exhaustion, indicating that additional signaling pathways are involved in T cell exhaustion.

Previous studies in cancer have suggested that GAS5 puts a “brake” on cell proliferation, thereby acting as a potential tumor suppressor in various cancers (46). Also, overexpression of GAS5 has been shown to increase apoptosis and reduce cell cycle progression in T cell lines and PBMCs (47). In contrast, low levels of GAS5 are often associated with cell proliferation and cancer metastasis (48). These findings in cancer cells seem to contradict our data showing that decreasing GAS5 in the CD4 T cells of PLHIV resulted in a dampened response in these prematurely aged cells while overexpressing GAS5 restored their dysregulated functions. As a multifunctional lncRNA, GAS5 plays crucial roles in many biological processes, including transcriptional regulation, by acting as a decoy or through promoting histone modifications or competing with endogenous RNA (ceRNA) to regulate various signaling pathways and biological functions (49). Thus, GAS5 expression and functions can be species-, cell-, or disease-specific. In addition, miR-21 is considered a cell activation marker and an oncogene, where its upregulation can cause an overgrowth of many cancers (50). In contrast, it has been shown to be a negative regulator of T cell activation. High expression of miR-21 in T lymphocytes can reduce activation marker (CD69, OX40, CD25, and CD127) and cytokine (IL-2, IFN-γ) gene expressions, and hence, induce T cell over-activation and dysfunction (51). Furthermore, it has been reported that upregulation of miR-21 level in the elderly drives proliferation of naïve T cells to a terminal phenotype instead of a long-lived memory phenotype, thus attenuating T cell responses; this strongly supports our findings, including TCF1 declines upon miR-21 silencing in this study. Previous studies have shown that miR-21 targets several pathways involved in cellular proliferation. The tumor-suppressor pathway–phosphatase and tension homolog (PTEN)/phosphoinositide 3-kinases (PI3K)–is a well-known target of miR-21 (52). Also, miR-21 has been shown to target the programmed cell death 4 (PDCD4)/p21 and Sprouty RTK Signaling Antagonist 1 (SPRY1)/Rapidly Accelerated Fibrosarcoma (RAF) signaling pathways (53, 54). Also, we and others have shown that chronic viral infection-induced inflammaging phenotypes result from deficiencies in Top 1/2α, ATM, hTERT, and TRF2, which correlate closely with the observed, aberrant CD4 T cell apoptosis and depletion. Here, we further demonstrate that a decrease in GAS5 level promotes the upregulation of miR-21 expression and that the GAS5-miR-21 axis may represent a key signaling pathway in controlling persistent activation, exhaustion, senescence, DNA damage, cell apoptosis, and cytokine dysregulation in CD4 T cells derived from PLHIV. Our data showed that GAS5 regulates CD4 T cell responses, not only through miR-21-mediated signaling but also via other undetermined pathways. In support of this notion, we showed that manipulating GAS5/miR-21 expressions or interactions during TCR activation can partially reverse CD4 T cell dysfunctions and apoptosis. These findings provide a novel strategy to improve immunotherapies and vaccine responses in the setting of human viral infection.

PLHIV on ART represent the major HIV-infected population in the era of ART. In this clinical setting, how the differential expression of GAS5 and miR-21 ultimately control CD4 T cell functions remains unknown. Given ART control of HIV replication leads to a very small proportion (one in a million) of PBMCs harboring HIV provirus (55), it is unlikely that HIV itself *per se* can cause GAS5 downregulation and miR-21 upregulation to compromise CD4 T cell functions. While data regarding GAS5 and miR-21 expression at day 0 (baseline level) are included in Figures 1A,B, we did not include this time point in the kinetic experiments due to limited cell volumes. However, our TCR kinetic stimulation results (Figures 1F–K) clearly indicate that GAS5 and miR-21 expressions are significantly regulated by T cell activation status. Notably, higher amounts of miR-21 and miR-146, along with Ago2, were detected in CD4 T cells from HIV and HS under TCR stimulation conditions, but with no significant differences between HIV and HS, indicating that the altered function of miR-21 in CD4 T cells is driven by GAS5 rather than Ago2. Since miRNAs form a duplex–with one strand preferentially loaded into AGO2 to generate a functional miRNA-induced silencing complex (miRISC)–it is likely that more miRNAs are loaded onto the Ago2-miRISC in CD4 T cells under TCR activation. Indeed, we and others have shown that ART-controlled PLHIV with no detectable viral replication can still exhibit an immune aging phenotype. We thus believe that the observed changes in GAS5 and miR-21 expression and dysregulations of CD4T cell functions in these virus-controlled PLHIV are likely induced by either immunologic scarring during early active viral infection or, perhaps more likely, by low-grade inflammation and persistent T cell activation during latent viral infection, or both. Our interpretation of the cumulative data would include the notion that CD4 T cells in PLHIV on ART exhibit an immune aging phenotype induced by a myriad of viral/host factors, including HIV reservoirs, that may secrete undetectable viral components, pro-inflammatory mediators, increased ROS levels, increased gut permeability and gut microbiota, coinfection with other pathogens (such as HBV, HCV, EBV, and CMV), ART regimens, associated malignancies, and social-related stresses (56–63), all of which may contribute to the failure to restore CD4 T cell homeostasis and/or functionality. These factors can lead to persistent, low-grade inflammation, causing T cell over-activation, exhaustion, senescence, apoptosis, and decreased proliferative potential, as our results suggest. Importantly, such inflammation-driven DNA damage promotes an inflammaging phenotype and exposes the immune system to unique challenges that could lead to CD4 T cell exhaustion, senescence, apoptosis, and homeostasis–a major driver of the increased incidences of infections, cancers, cardiovascular, and neurodegenerative diseases, similar to that observed in the elderly. Therefore, this premature immune aging process puts PLHIV at greater risk of morbidity and mortality.

It should be pointed out that, while the decrease in GAS5 and increase in miR-21 can explain both DNA damage and cell apoptosis, their presence can also result in both overwhelming cell death in acute infection and immune tolerance or immune suppression in chronic infection. Nevertheless, our findings identify, for the first time, the role of the GAS5-miR-21 axis in CD4 T cell dysregulation in PLHIV and shed light on the molecular aspects of immunomodulation during human viral infections. Given the broad regulatory effects of both GAS5 and miR-21, the decline in GAS5 and increase in miR-21 may impair diverse cellular functions during chronic HIV infection. Thus, interrupting this GAS5-miR21 axis may restore CD4 T cell homeostasis and competency during latent HIV infection and prevent premature CD4 T cell aging or immune senescence. This study reveals a novel molecular mechanism underlying CD4 T cell aging and provides a new strategy to develop innovative approaches to correct the aberrant immunopathology, to avoid the untoward consequences of immune senescence and to improve immunotherapy as well as vaccine responses against human viral diseases.

## Data Availability Statement

The original contributions presented in the study are included in the article/supplementary material, further inquiries can be directed to the corresponding author/s.

## Ethics Statement

The studies involving human participants were reviewed and approved by East Tennessee State University and James H. Quillen VA Medical Center. The patients/participants provided their written informed consent to participate in this study.

## Author Contributions

LNTN performed most of the experiments. LNN, JZhao, MS, XD, DC, SK, BC, and ZLu participated in some experiments. XW and ZM provided technical support. SN, ME, LW, JZhang, ZLi, and JM offered intellectual input for troubleshooting and discussion of the findings. ZY supervised the project and wrote the manuscript, with the help of LNTN, LNN, JZhang, MS, XD, DC, SK, BC, ZLu, JZhang, ZLi, ZM, XW, ME, SN, LW, and JM. All authors contributed to the article and approved the submitted version.

## Conflict of Interest

The authors declare that the research was conducted in the absence of any commercial or financial relationships that could be construed as a potential conflict of interest.
